# Efficacy of Lenvatinib as Second-Line Therapy After Atezolizumab Plus Bevacizumab for Hepatocellular Carcinoma

**DOI:** 10.3390/curroncol33030159

**Published:** 2026-03-11

**Authors:** Daichi Takizawa, Hirotaka Arai, Mitsuhiko Shibasaki, Yuki Tamura, Satoru Kakizaki, Takeshi Hatanaka, Atsushi Naganuma, Takashi Ueno, Toru Fukuchi, Masashi Namikawa, Satoshi Takakusagi, Shuichi Saito, Takayoshi Suga, Hiroki Tojima, Yuichi Yamazaki, Toshio Uraoka

**Affiliations:** 1 Department of Gastroenterology, Maebashi Red Cross Hospital, Maebashi 371-0811, Japan; takizawa.biz@gmail.com (D.T.);; 2 Department of Clinical Research, NHO Takasaki General Medical Center, Takasaki 370-0829, Japan; 3 Department of Gastroenterology, Gunma Saiseikai Maebashi Hospital, Maebashi 371-0821, Japan; 4 Department of Gastroenterology, NHO Takasaki General Medical Center, Takasaki 370-0829, Japan; 5 Department of Internal Medicine, Isesaki Municipal Hospital, Isesaki 372-0817, Japan; 6 Department of Internal Medicine, Kiryu Kosei General Hospital, Kiryu 376-0024, Japan; 7 Department of Gastroenterology, Subaru Health Insurance Society Ota Memorial Hospital, Ota 373-8585, Japan; 8 Department of Gastroenterology and Hepatology, Kusunoki Hospital, Fujioka 375-0024, Japan; 9 Department of Gastroenterology, Public Tomioka General Hospital, Tomioka 370-2393, Japan; 10 Department of Gastroenterology, NHO Shibukawa Medical Center, Shibukawa 377-0280, Japan; 11 Department of Gastroenterology and Hepatology, Gunma University Graduate School of Medicine, Maebashi 371-8511, Japan

**Keywords:** lenvatinib, atezolizumab, bevacizumab, second-line, hepatocellular carcinoma

## Abstract

**Background:** Atezolizumab plus bevacizumab (ATZ/BEV) is widely used as first-line therapy for advanced hepatocellular carcinoma (HCC); however, optimal subsequent treatment after ATZ/BEV failure remains unclear. **Methods:** Between October 2020 and August 2024, 165 patients with unresectable HCC treated with first-line ATZ/BEV were retrospectively analyzed. After excluding patients with insufficient follow-up, outcomes were compared between those who received lenvatinib (LEN) as second-line therapy (*n* = 49) and those who did not (*n* = 95). **Results:** Median overall survival (OS) was significantly longer in the LEN group than in the non-LEN group (20.9 vs. 8.47 months, *p* < 0.01). LEN administration was independently associated with improved OS (hazard ratio 0.48), and this benefit remained significant after inverse probability weighting adjustment (adjusted hazard ratio 0.50). Among LEN-treated patients, a lower albumin–bilirubin score before ATZ/BEV and a total LEN dose ≥ 400 mg were independent prognostic factors. **Conclusions:** Lenvatinib as second-line therapy after ATZ/BEV was associated with improved survival in unresectable HCC. Preservation of liver function and the ability to maintain adequate lenvatinib exposure were associated with favorable outcomes, likely reflecting baseline prognosis and treatment feasibility rather than lenvatinib-specific predictive factors.

## Simple Summary

Atezolizumab plus bevacizumab is used as first-line treatment for advanced hepatocellular carcinoma, but evidence regarding subsequent second-line therapy remains insufficient. This multicenter collaborative study retrospectively examined cases treated with lenvatinib as second-line therapy. Results showed that patients receiving lenvatinib had improved survival compared with those who did not receive it. Additionally, factors contributing to prognosis in patients receiving lenvatinib as second-line therapy included hepatic reserve before first-line treatment and a total lenvatinib dose of 400 mg or more. Maintaining hepatic reserve through appropriate dose management was considered important. These findings provide clinically useful information regarding the feasibility of sequential systemic therapy following atezolizumab plus bevacizumab treatment and highlight factors associated with preserved hepatic reserve and treatment continuation in routine clinical practice.

## 1. Introduction

Hepatocellular carcinoma (HCC) remains a critical global health challenge, ranking as the sixth most common cancer and the third leading cause of cancer-related mortality worldwide [1,2]. The landscape of systemic therapy for advanced HCC has evolved rapidly over the past two decades. The paradigm shift began in 2008 with the SHARP trial, which established sorafenib as the first effective systemic treatment [3]. This was followed in 2018 by the REFLECT trial, where lenvatinib (LEN) demonstrated non-inferiority to sorafenib with a notably higher objective response rate [4]. In 2020, the IMbrave150 trial revolutionized first-line treatment by showing the superiority of atezolizumab plus bevacizumab (ATZ/BEV), an immune checkpoint inhibitor (ICI) and vascular endothelial growth factor (VEGF) inhibitor combination, over sorafenib [5].

The therapeutic armamentarium continues to expand with the introduction of new ICI-based regimens. The HIMALAYA trial (2022) introduced the STRIDE regimen (tremelimumab plus durvalumab) [6]. More recently, the phase 3 CheckMate-9DW trial (2025) demonstrated the efficacy of nivolumab plus ipilimumab as a first-line option, further diversifying the immunotherapy-based landscape [7]. Despite these advancements, ATZ/BEV remains a standard first-line therapy, yet there is no established consensus on the optimal second-line treatment after its failure.

Lenvatinib has emerged as a promising candidate for second-line therapy. Recent real-world studies have reported favorable outcomes for LEN following ATZ/BEV treatment [8,9,10]. A key biological rationale for this sequence is the potential “pseudo-combination effect,” where the lingering effects of ICI therapy may interact synergistically with the subsequent VEGFR inhibition provided by LEN [11]. Furthermore, modern analytical approaches, such as the target trial emulation study, have begun to use advanced causal inference to compare the effectiveness of ICIs versus LEN in various clinical contexts, highlighting that treatment sequencing is a critical determinant of long-term survival [12].

A major challenge in sequential therapy is the preservation of hepatic reserve. The prognosis of HCC patients is inextricably linked to liver function, often assessed by the albumin–bilirubin (ALBI) score. Maintenance of liver function during first-line ATZ/BEV is essential to ensure that patients remain eligible for second-line systemic agents [13,14]. In real-world practice, achieving a balance between therapeutic efficacy and safety through appropriate dose management is vital, as cumulative drug exposure may be a significant prognostic factor [15].

Therefore, we conducted this multicenter study across 10 institutions to evaluate the real-world efficacy and prognostic factors of LEN as second-line therapy after ATZ/BEV failure. This study specifically focuses on the impact of pre-treatment hepatic reserve and cumulative LEN dosage to provide clinical evidence for optimal sequential treatment strategies in patients with unresectable HCC.

## 2. Methods

### 2.1. Patients

**Figure 1:**
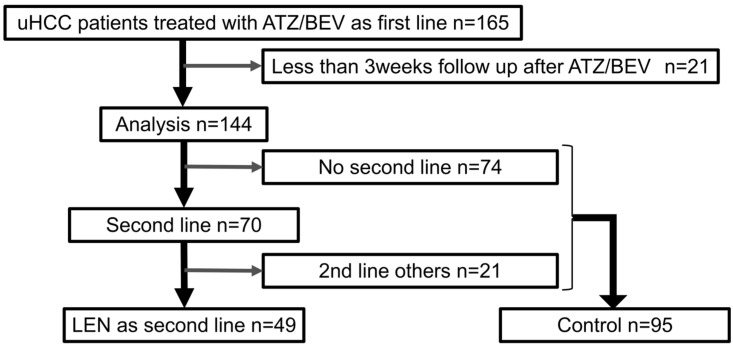
Patient flowchart of the study population.

This retrospective multicenter study analyzed 165 patients with unresectable HCC who received ATZ/BEV as first-line therapy at 10 institutions in Gunma Prefecture, Japan, between October 2020 and August 2024. To evaluate prognosis after ATZ/BEV, patients with less than 3 weeks of follow-up after the final ATZ/BEV dose were excluded. Among 70 patients who received second-line treatment, 49 were treated with LEN. Twenty-one patients received agents other than LEN, and 74 patients did not receive any second-line therapy (Figure 1).

A total of 165 patients with unresectable hepatocellular carcinoma (HCC) who received atezolizumab plus bevacizumab (ATZ/BEV) as first-line therapy were screened. Among them, 70 patients received second-line systemic therapy. After exclusion of patients with less than 3 weeks of follow-up after ATZ/BEV (*n* = 21), 144 patients were included in the final analysis. The non-LEN group was heterogeneous and included patients who received other systemic therapies as well as those who did not receive further treatment. Therefore, this group should be interpreted as representing real-world post-progression management rather than a uniform treatment comparator.

Patients positive for anti-HCV antibodies were classified as having HCV-related HCC. HBV-related HCC included patients positive for HBsAg or HBV-related antibodies. Metabolic dysfunction–associated steatohepatitis (MASH), previously referred to as NASH, and alcohol-related liver disease were classified as non-B non-C (NBNC) [16,17]. Patients with autoimmune diseases were not treated with ATZ/BEV.

Liver function was assessed using both the Child–Pugh classification and the albumin–bilirubin (ALBI) score. Modified ALBI grades subdivided grade 2 into 2a and 2b [18]. HCC was diagnosed based on elevated AFP and/or PIVKA-II levels, dynamic CT or EOB-MRI findings, and/or histopathological confirmation [19,20,21]. Tumor staging was assessed using the BCLC staging system [22].

Overall survival (OS) was compared between the LEN and non-LEN groups using the Kaplan–Meier method and log-rank test. Propensity score matching (PSM) analysis was also performed. Objective response rate (ORR), disease control rate (DCR), progression-free survival (PFS), and prognostic factors (including ALBI score, tumor size, and total LEN dose) were evaluated using multivariate analyses.

### 2.2. Treatment and Evaluation

Atezolizumab (1200 mg) and bevacizumab (15 mg/kg) were administered intravenously every three weeks [5]. Lenvatinib was administered at 12 mg/day for patients weighing ≥60 kg and 8 mg/day for those <60 kg, with dose adjustments based on liver function, comorbidities, patient condition, and adverse events [4]. Treatment was continued until withdrawal of consent, death, disease progression, or unacceptable toxicity.

Treatment strategies, including second-line therapy and the use of liver-directed treatments, were typically discussed in multidisciplinary tumor boards at each institution. Final treatment decisions were made by the responsible physicians based on these discussions and individual patient factors. In cases where liver-directed therapies such as transarterial chemoembolization, ablation, or radiotherapy were considered feasible, treatment selection was determined individually through multidisciplinary discussion. Patients who underwent local therapy after response or who were deemed unsuitable for further systemic treatment were managed accordingly.

Adverse events were evaluated according to CTCAE version 5.0 [23]. Dose initiation, reduction, or discontinuation of LEN was determined by the treating physician. This study was conducted in accordance with the Declaration of Helsinki and Japanese clinical research guidelines after appropriate institutional approval.

Tumor response was evaluated using modified RECIST (mRECIST) criteria [24] and classified as complete response (CR), partial response (PR), stable disease (SD), or progressive disease (PD). Tumor markers were assessed every 3–6 weeks after initiation of ATZ/BEV, and imaging evaluations were performed approximately every 3 months using dynamic CT or EOB-MRI. The choice between CT and MRI was made at the discretion of the treating physicians based on institutional practice, image quality, and clinical necessity, with MRI often used for further characterization when CT findings were inconclusive. Similar intervals were used for LEN assessment. PFS was determined based on mRECIST.

### 2.3. Statistical Analysis

Statistical analyses included Student’s *t*-test, Welch’s *t*-test, Mann–Whitney U test, Wilcoxon signed-rank test, Cox proportional hazards regression, log-rank test, and Kaplan–Meier analysis. Median values were presented with ranges. A *p* value < 0.05 was considered statistically significant. Covariate balance before and after inverse probability weighting was evaluated using standardized mean differences (SMDs), with values < 0.1 considered acceptable. All analyses were performed using Easy-R version 1.70 (Saitama Medical Center, Jichi Medical University, Saitama, Japan) [25].

## 3. Results

Baseline characteristics are shown in Table 1. The LEN and non-LEN groups included 49 and 95 patients, respectively. Median age was 72 vs. 75 years, and males accounted for 34 (69.4%) vs. 69 (72.6%) patients. In the non-LEN group, 21 patients received second-line systemic therapy, whereas 74 patients did not (Supplementary Table S1). Among the 21 patients, second-line treatments included sorafenib (*n* = 6), tremelimumab plus durvalumab (*n* = 10), durvalumab (*n* = 2), ramucirumab (*n* = 1), cabozantinib (*n* = 1), and other regimens (*n* = 1). Seventy-four patients did not receive second-line therapy. Among patients who did not receive second-line systemic therapy, some were managed with local treatments or best supportive care according to clinical condition. BMI was significantly higher in the LEN group (24.1 vs. 23.4; *p* = 0.030). ALBI score was better in the LEN group (−2.57 vs. −2.27; *p* < 0.01), and maximum tumor diameter was smaller (25.5 mm vs. 40 mm; *p* = 0.032). Diabetes and hypertension were not significantly different between groups, but hyperlipidemia was more common in the LEN group (28.6% vs. 11.6%; *p* = 0.019).

**Figure 2:**
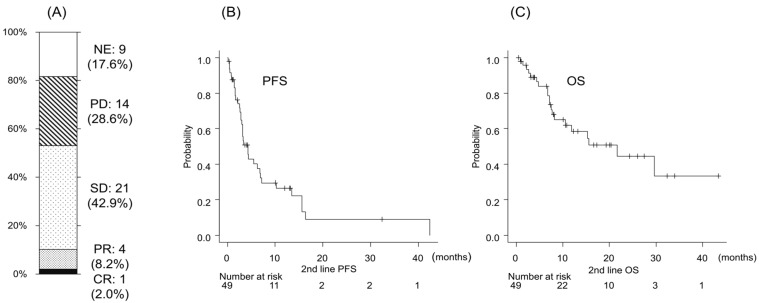
Clinical outcomes of patients treated with second-line lenvatinib. (**A**) Best overall response to lenvatinib according to mRECIST criteria, including complete response (CR), partial response (PR), stable disease (SD), progressive disease (PD), and not evaluable (NE). Kaplan–Meier curves showing (**B**) progression-free survival (PFS) and (**C**) overall survival in patients who received lenvatinib as second-line therapy after ATZ/BEV failure. Median PFS was 4.27 (95% CI: 2.86–6.80) months and median OS was 21.7 (95% CI: 8.18–NA). PFS and OS were calculated from the date of lenvatinib initiation.

The objective response rate and disease control rate of second-line LEN were 10.2% and 53.1%, respectively (CR:PR:SD:PD = 1:4:21:14, NE = 9). Median PFS was 4.27 months, while median OS was 21.7 months, which appeared favorable compared with previously reported outcomes of second-line lenvatinib [8,9,10] (Figure 2). Given the discrepancy between PFS and OS, OS was considered the primary outcome of clinical relevance in this study.

Data are presented as median [range] or number (%), as appropriate. BMI, body mass index; ALBI, albumin–bilirubin; DM, diabetes mellitus; HL, hyperlipidemia; HT, hypertension; BCLC, Barcelona Clinic Liver Cancer; AFP, alpha-fetoprotein; PIVKA-II, protein induced by vitamin K absence or antagonist-II.

Adverse events occurred in 43 patients (87.8%), and treatment discontinuation due to adverse events occurred in 18 patients. Proteinuria was the most frequent adverse event, followed by anorexia (Table 2). Dose reduction was required in a substantial proportion of patients, and grade ≥ 3 adverse events were not uncommon; however, treatment continuation was feasible in many cases, suggesting that lenvatinib could be administered with appropriate dose management even in patients with compromised liver function.

**Figure 3:**
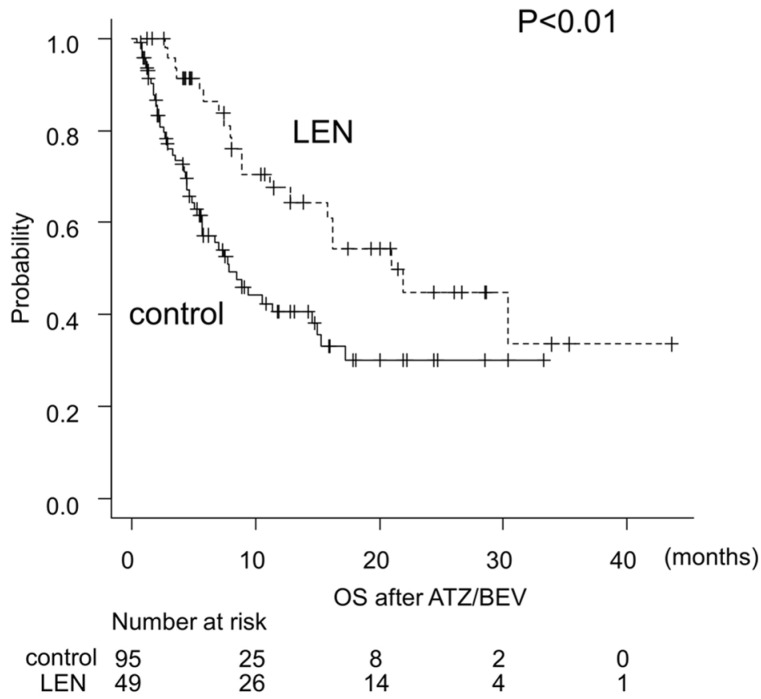
Overall survival in patients with and without second-line lenvatinib treatment. Kaplan–Meier curves comparing overall survival between patients who received lenvatinib as second-line therapy and those who did not receive lenvatinib. Median OS was 20.9 (95% CI: 12.7–NA) months in the LEN group and 8.47 (95% CI: 5.61–14.6) months in the non-LEN group (log-rank test, *p* < 0.01). OS was calculated from the end of ATZ/BEV treatment.

During the observation period, a total of 69 deaths occurred in the overall cohort, including 20 deaths in the LEN group. Overall survival from the end of ATZ/BEV was significantly longer in the LEN group than in the non-LEN group according to the log-rank test (*p* < 0.01) (Figure 3). This survival benefit was also confirmed by Cox proportional hazards analysis (hazard ratio [HR], 0.48; *p* < 0.01). Furthermore, this association remained robust after inverse probability weighting (IPW) adjustment for the four baseline variables that showed significant differences between the groups. (HR 0.501, *p* = 0.01) (Table 3). Standardized mean differences were substantially reduced after weighting. All measured covariates, including ALBI score, showed SMD values below the commonly accepted threshold of 0.1, indicating adequate balance between the groups.

Overall survival stratified by second-line treatment is shown in Supplementary Figure S1. Patients who received second-line therapy demonstrated significantly improved OS compared with those who did not receive second-line therapy (*p* < 0.01). No significant difference in OS was observed between LEN and the other agents.

In univariate analysis, prognostic factors of LEN group included pre-ATZ/BEV ALBI score, absence of portal vein tumor thrombosis, total LEN dose ≥ 400 mg, and tumor diameter ≤ 30 mm. Multivariate analysis identified pre-ATZ/BEV ALBI score and total LEN dose as independent prognostic factors (Table 4).

**Figure 4:**
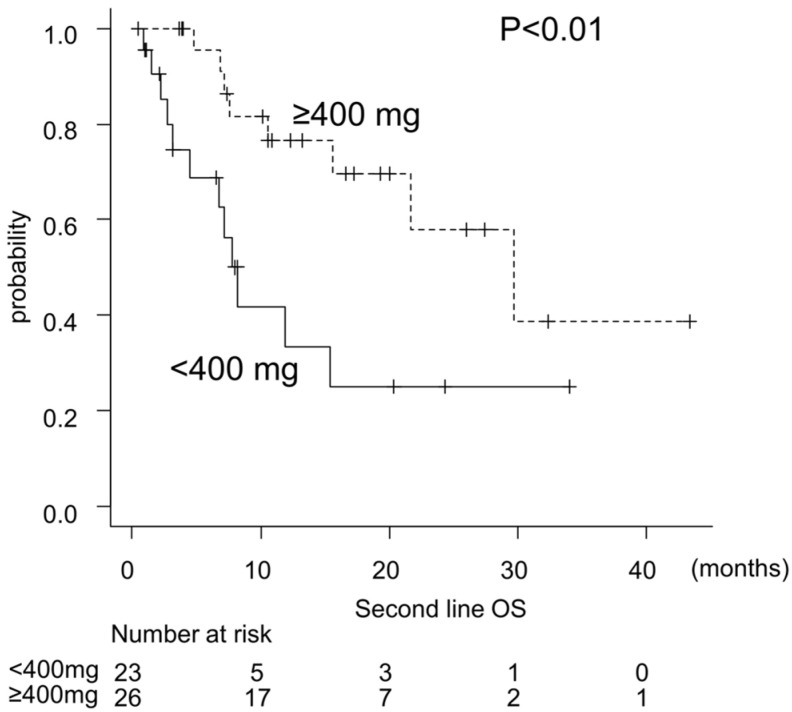
Impact of total lenvatinib dose on overall survival. Kaplan–Meier curves of overall survival stratified by total lenvatinib dose (≥400 mg vs. <400 mg) among patients treated with lenvatinib as second-line therapy. Patients who received a total dose of ≥400 mg (median 29.7, 95% CI: 15.6–NA months) showed significantly longer OS than <400 mg (median 7.72, 95% CI: 3.12–15.3 months) (log-rank test, *p* < 0.01).

Dose reduction was initiated in 69.3% of patients without worsening prognosis, and discontinuation due to adverse events was less frequent. Total LEN dose did not differ between patients with and without dose reduction, and outcomes remained favorable in patients receiving ≥400 mg (Figure 4).

**Figure 5:**
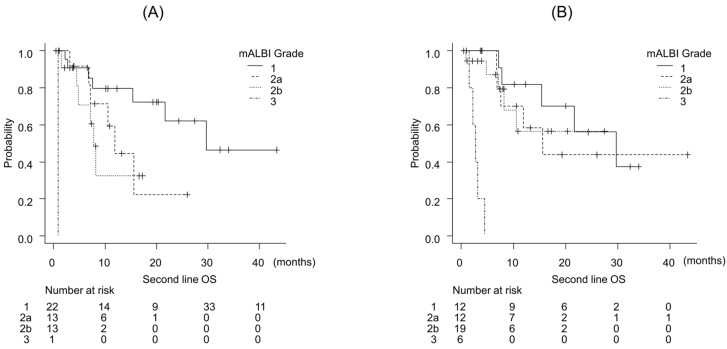
Prognostic impact of liver function on overall survival in patients treated with lenvatinib. Kaplan–Meier curves of overall survival stratified by modified albumin–bilirubin (ALBI) grade assessed (**A**) before initiation of atezolizumab plus bevacizumab and (**B**) before initiation of lenvatinib.

ALBI scores significantly worsened from before ATZ/BEV to before LEN initiation (*p* < 0.01). Survival after LEN initiation was better stratified by pre-ATZ/BEV ALBI grade than by pre-LEN ALBI grade (Figure 5).

## 4. Discussion

This study demonstrated improved survival outcomes in patients who received lenvatinib as second-line therapy after ATZ/BEV. The non-LEN group included patients who received other second-line treatments or no second-line therapy. Furthermore, because a variety of drugs were used for second-line treatment and the number of cases was small, it was difficult to compare second-line treatment drugs. There were also various reasons for not undergoing second-line treatment, including cases of decreased liver function due to progression of liver cancer or side effects, cases of marked response, and transition to local therapy after response.

Importantly, the non-LEN group in this study was not a uniform comparator but reflected heterogeneous real-world management after ATZ/BEV failure, including other systemic therapies, local treatments, and best supportive care. Because treatment allocation after progression is strongly influenced by liver function, tumor burden, and patient condition, survival differences between groups should not be interpreted as reflecting the intrinsic superiority of lenvatinib alone. Rather, the results may partly reflect the overall feasibility of continuing active treatment.

Treatment selection after ATZ/BEV failure in routine practice involves not only systemic therapy but also consideration of liver-directed treatments when feasible. In the present cohort, decisions regarding second-line therapy were made individually based on tumor progression pattern, hepatic reserve, and overall patient condition. Therefore, our results should be interpreted within the context of real-world multidisciplinary treatment decision-making rather than a fixed treatment algorithm.

Given the heterogeneity of these factors, we focused our study on the effectiveness of LEN after ATZ/BEV. The outcomes were comparable not only to previous reports of second-line LEN after ATZ/BEV but also to the median survival time reported in the REFLECT trial of first-line LEN [4]. In addition to Japanese cohort studies, several international real-world analyses have also explored treatment sequencing after ATZ/BEV failure. Although patient populations and available drugs differ across regions, these studies similarly suggest that maintaining hepatic reserve and enabling continued systemic therapy are key determinants of long-term outcomes. Our findings are broadly consistent with these observations and provide additional data from a multicenter regional cohort.

Hiraoka et al. [8] reported favorable outcomes of second-line LEN after ATZ/BEV, particularly in patients with preserved liver function. In our study, prognosis after LEN was better stratified by ALBI score before ATZ/BEV rather than before LEN, suggesting that post-ATZ/BEV ALBI deterioration may partly reflect transient treatment-related effects rather than true hepatic reserve.

Although the response rate and PFS were lower than in previous studies [8,9,10], OS was longer (21.7 months). This discrepancy between PFS and OS suggests that survival benefit may be driven not solely by tumor response, but also by treatment tolerability, maintenance of liver function, and subsequent therapies. Appropriate dose adjustment enabled treatment continuation without compromising efficacy, consistent with previous reports [10]. Total lenvatinib dose remained associated with survival even among patients with disease control. However, this association should be interpreted with caution because of the potential for reverse causation; patients who survive longer are inherently more likely to receive higher cumulative doses. Therefore, cumulative lenvatinib dose may partly reflect treatment duration and patient survival rather than a direct causal effect of dose itself. In addition, the relationship between cumulative lenvatinib dose and survival may be influenced by time-dependent bias. Patients who tolerate treatment well and maintain clinical stability are more likely to continue therapy and accumulate higher doses. Thus, cumulative dose should be interpreted primarily as a surrogate of treatment feasibility and tolerability rather than as a direct predictor of therapeutic efficacy. Prospective studies incorporating time-dependent analyses would be required to clarify whether cumulative dose itself has an independent causal impact on prognosis.

Importantly, the factors identified in this study should be interpreted primarily as prognostic indicators rather than lenvatinib-specific predictive markers. Preserved hepatic reserve before first-line therapy likely reflects the overall capacity to undergo sequential systemic treatment, while cumulative lenvatinib dose may represent treatment tolerability and continuation rather than intrinsic drug sensitivity. Therefore, these findings should not be interpreted as suggesting that these factors can be used to select patients specifically for lenvatinib, but rather as indicators of patients more likely to benefit from ongoing systemic therapy in general.

The persistence of immune checkpoint inhibitor effects and the potential “pseudo-combination effect” of VEGFR inhibitors after immunotherapy may have contributed to the efficacy of LEN [8,9]. Although some reports have suggested that sequential immune checkpoint inhibitor therapy may show limited efficacy [26], the available evidence remains largely retrospective and inconclusive. Moreover, the landscape of second-line immunotherapy for HCC continues to evolve, with emerging regimens such as STRIDE or nivolumab plus ipilimumab being actively investigated.

Recent real-world analyses using advanced causal inference methods have also compared outcomes between immunotherapy and lenvatinib in advanced HCC. For example, a target trial emulation study by Ahn et al. reported broadly comparable effectiveness between immunotherapy and lenvatinib in certain clinical contexts [12], highlighting that the optimal sequencing of systemic therapies remains uncertain. In this context, selecting a mechanistically distinct agent such as lenvatinib may represent a reasonable option, particularly in patients requiring rapid disease control. Future prospective studies are needed to clarify the optimal sequencing of immunotherapy and targeted agents after ATZ/BEV.

Safety is a particularly important consideration in patients receiving second-line systemic therapy after ATZ/BEV, as hepatic reserve is often impaired at this stage. In the present study, dose reductions were frequently required, and grade ≥ 3 adverse events were observed in a notable proportion of patients. However, treatment discontinuation due to toxicity was relatively limited, suggesting that lenvatinib could be administered with careful dose adjustment and monitoring. These findings are consistent with previous real-world studies indicating that tolerability and dose management are key determinants of treatment feasibility in sequential therapy for HCC.

This study has several limitations. First, because the analysis was retrospective, treatment allocation was not randomized and selection bias is unavoidable. Patients in the LEN group tended to have relatively preserved liver function and smaller tumor burden at baseline, suggesting that physicians may have preferentially selected candidates considered suitable for further systemic therapy. Although IPW-adjusted analyses were performed to reduce baseline imbalances, residual confounding cannot be completely excluded. Second, because the number of patients included in the analysis was relatively small, the non-LEN group included not only patients who did not receive second-line therapy but also patients who received other systemic agents, which may have introduced heterogeneity in treatment exposure. Third, the association between cumulative lenvatinib dose and improved survival should be interpreted cautiously, as it may be influenced by time-dependent bias and reverse causation; patients who survive longer are inherently more likely to accumulate higher drug exposure. Future studies with larger patient populations and prospective designs are needed to clarify optimal treatment sequencing after ATZ/BEV and to better evaluate the independent impact of lenvatinib exposure on prognosis. Therefore, the present findings should be interpreted as hypothesis-generating rather than definitive evidence of treatment superiority.

## 5. Conclusions

Lenvatinib as second-line therapy after atezolizumab plus bevacizumab was associated with improved survival in this cohort, although the potential influence of treatment selection bias should be considered. Preservation of liver function and appropriate dose management—together with careful safety monitoring—were important contributors to favorable outcomes. These factors likely reflect baseline prognosis and treatment feasibility rather than lenvatinib-specific predictive markers, and may help identify patients suitable for continued sequential systemic therapy.

## Figures and Tables

**Table 1:** Baseline characteristics of patients with and without second-line lenvatinib treatment.

Factors	LEN *n* = 49	Non-LEN *n* = 95	*p*
Age, years	72.0 [48.0, 91.0]	75.0 [48.0, 90.0]	0.134
Gender, Male (%)	34 (69.4)	69 (72.6)	0.700
BMI, kg/m^2^	24.1 [17.9, 40.7]	23.4 [12.8, 33.7]	0.030
Etiology, HBV/HCV/Both/NBNC (%)	3/20/3/23 (6.1/40.8/6.1/46.9)	11/43/1/40 (11.6/45.3/1.1/42.1)	0.242
Child-Pugh grade A (%)	42(85.7)	69 (72.6)	0.095
ALBI score	−2.57 [−3.10, −1.36]	−2.27 [−3.07, −1.03]	<0.01
Protein in urine (%)	10 (20.4)	19 (20.0)	1.000
DM (%)	25 (51.0)	46 (48.4)	0.861
HL (%)	14 (28.6)	11 (11.6)	0.019
HT (%)	31 (63.3)	53 (55.8)	0.476
BCLC stage, A/B/C (%)	4/22/21 (8.5/46.8/44.7)	11/29/55 (11.6/30.5/57.9)	0.161
TNM stage, 2/3/4A/4B (%)	14/15/6/13 (29.2/31.2/12.5/27.1)	20/37/9/29 (21.1/38.9/9.5/30.5)	0.593
Number of tumor ≥ 4 (%)	30 (61.2)	54 (56.8)	0.722
Tumor diameter, mm	25.5 [5.0, 131.0]	40.0 [5.0, 190.0]	0.032
Up-to-7 out (%)	25 (53.2)	59 (64.1)	0.271
Portal vein invasion (%)	7 (14.3)	23 (24.2)	0.198
AFP, ng/mL	51.86 [1.7, 444,587.2]	77.90 [2.0, 200,000.0]	0.540
PIVKA-II, mAU/mL	118.8 [10.1, 88,500.0]	201.0 [11.0, 297,033.0]	0.272

**Table 2:** Adverse events of second-line lenvatinib therapy.

CTCAE Version 5.0	Any Grade	Grade ≥ 3
Protein in urine	19 (38.8%)	5 (10.2%)
Appetite loss	16 (32.7%)	6 (12.2%)
General fatigue	5 (10.2%)	1 (2.0%)
Hypertension	7 (14.3%)	0
Other adverse events		diarrhea 2, edema 2, ascites 1, cerebral infarction 1, liver dysfunction 2

Data are presented as number (%). CTCAE, Common Terminology Criteria for Adverse Events.

**Table 3:** Cox proportional hazards analysis of lenvatinib for overall survival.

	Hazard Ratio (95% CI)	*p*
Unadjusted	0.481 (0.284–0.813)	<0.01
IPW adjusted (BMI, ALBI, HL, tumor diameter)	0.501 (0.296–0.848)	0.01

CI, confidence interval; IPW, inverse probability weighting; BMI, body mass index; ALBI, albumin–bilirubin; HL, hyperlipidemia.

**Table 4:** Univariate and multivariate analyses of prognostic factors for overall survival in patients treated with second-line lenvatinib.

	Univariate Analysis		Multivariate Analysis	
	HR (95% CI)	*p*	HR (95% CI)	*p*
ALBI Grade 1 (pre-ATZ/BEV)	0.33 (0.12–0.91)	0.031	0.28 (0.099–0.79)	0.016
ALBI Grade1 (pre-LEN)	0.51 (0.18–1.45)	0.21		
Viral etiology	0.47 (0.19–1.14)	0.093		
Age ≥ 75	1.79 (0.74–4.32)	0.19		
Tumor diameter > 30 mm	3.17 (1.20–8.38)	0.02	1.95 (0.62–6.04)	0.25
Number of tumor ≥ 4	1.13 (0.41–3.11)	0.82		
Portal vein invasion	3.70 (1.27–10.78)	0.017	1.53 (0.54–4.35)	0.43
LEN ≥ 400 mg	0.31 (0.13–0.77)	0.012	0.27 (0.11–0.67)	<0.01
Disease control	0.69 (0.28–1.67)	0.41		

Variables with *p* < 0.10 in univariate analysis were included in the multivariate model. HR, Hazard ratio; CI, confidence interval; ALBI, albumin–bilirubin; LEN, lenvatinib.

## Data Availability

De-identified patient data used in the analyses are not publicly available due to privacy and ethical restrictions but will be made available to researchers upon written request to the corresponding author for scientific purposes.

## Supplementary Materials

The following supporting information can be downloaded at: https://www.mdpi.com/article/10.3390/curroncol33030159/s1, Figure S1: Overall survival stratified by second-line treatment; Table S1: Baseline characteristics of patients stratified by second-line treatment.

## Abbreviations

The following abbreviations are used in this manuscript:

ATZ/BEV	Atezolizumab plus bevacizumab
LEN	lenvatinib
