# Supplementary material for: Efficacy of Lenvatinib as Second-Line Therapy After Atezolizumab Plus Bevacizumab for Hepatocellular Carcinoma
Source: Curr Oncol. 2026 Mar 11;33(3):159. doi: 10.3390/curroncol33030159 (PMC13025887; doi:10.3390/curroncol33030159)
Supplement: Supplementary file 1 [file curroncol-33-00159-s001.zip › curroncol-4165550-supplementary.pdf]

Supplemental Table S1. Baseline characteristics of patients stratified by second-line treatment.

Factors	LEN n=49	Other 2nd-line n=21	No 2nd-line n=74	*P*
Age, years	72.0 [48.0, 91.0]	75.00 [63.0, 88.0]	76.0 [48.0, 90.0]	0.288
Gender, Male (%)	34 (69.4)	18 (85.7)	51 (68.9)	0.296
BMI, kg/m^2	24.14 [17.90, 40.70]	24.17 [12.83, 29.76]	22.61 [17.71, 33.53]	0.062
Etiology, HBV/HCV/Both/NBNC (%)	3/20/3/23 (6.1/40.8/6.1/46.9)	2/13/0/6 (9.5/61.9/0/28.6)	9/30/1/34 (12.2/40.5/1.4/45.9)	0.289
ChildA (%)	42(85.7)	17 (81.0)	52 (70.3)	0.123
ALBI score	-2.57 [-3.10, -1.36]	-2.28 [-3.07, -1.30]	-2.25 [-3.03, -1.03]	<0.01
Protein in urine (%)	10 (20.4)	6 (28.6)	13 (17.6)	0.539
DM (%)	25 (51.0)	10 (47.6)	36 (48.6)	0.954
HL (%)	14 (28.6)	2 (9.5)	9 (12.2)	0.037
HT (%)	31 (63.3)	13 (61.9)	40 (54.1)	0.56
BCLC stage, A/B/C (%)	4/22/21 (8.5/46.8/44.7)	4/7/10 (19.0/33.7/47.6)	7/22/45 (9.5/29.7/60.8)	0.228
TNM stage, 2/3/4A/4B (%)	14/15/6/13 (29.2/31.2/12.5/27.1)	7/8/0/6 (33.3/38.1/0/28.6)	13/29/9/23 (17.6/39.2/12.2/31.1)	0.438
Number of tumor ≥ 4 (%)	30 (61.2)	12 (57.1)	42 (56.8)	0.88
Tumor diameter, mm	25.5 [5.0, 131.0]	28.00 [9.00, 126.00]	45.00 [5.00, 190.00]	0.034
Up-to-7 out (%)	25 (53.2)	12 (57.1)	47 (66.2)	0.348
Portal vein invasion (%)	7 (14.3)	4 (19.0)	19 (25.7)	0.306
AFP, ng/ml	51.86 [1.7, 444587.2]	14.90 [2.00, 4249.00]	107.47 [2.00, 200000.00]	0.374
PIVKA-II, mAU/ml	118.8 [10.1, 88500.0]	120.00 [13.00, 25490.00]	280.00 [11.00, 297033.00]	0.238

Supplementary Figure S1. Overall survival stratified by second-line treatment

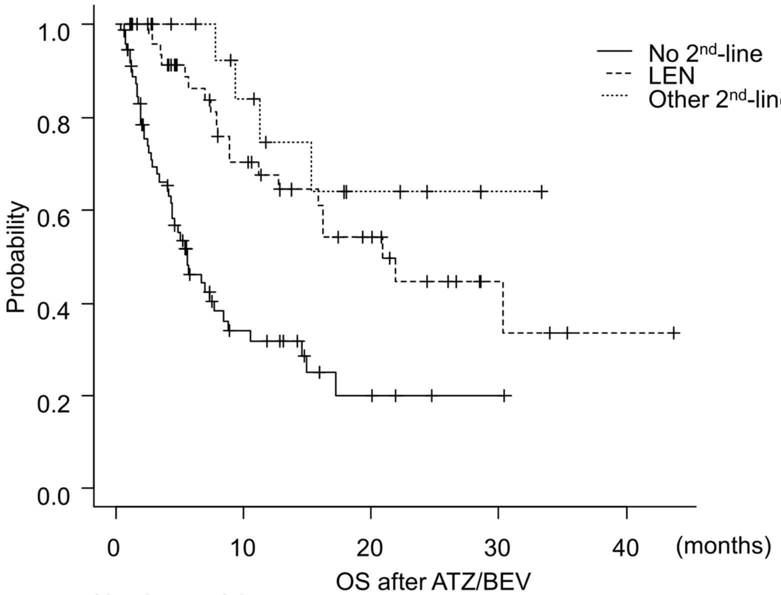

Number at risk

No 2L	74	15	4	1	0
LEN	49	26	14	4	1
Other 2L	21	10	4	1	0
